# Characterization of *Clostridium Perfringens* Isolates Collected from Three Agricultural Biogas Plants over a One-Year Period

**DOI:** 10.3390/ijerph17155450

**Published:** 2020-07-29

**Authors:** Lorine Derongs, Céline Druilhe, Christine Ziebal, Caroline Le Maréchal, Anne-Marie Pourcher

**Affiliations:** 1 INRAE, OPAALE Research Unit, CS 64427, F-35044 Rennes, France; lorine.derongs@inrae.fr (L.D.); celine.druilhe@inrae.fr (C.D.); christine.ziebal@inrae.fr (C.Z.); 2 ANSES, Ploufragan-Plouzané-Niort Laboratory, Hygiene and Quality of Poultry and Pig Products Unit, BP53, F-22440 Ploufragan, France; caroline.lemarechal@anses.fr

**Keywords:** *C. perfringens*, mesophilic anaerobic digestion, manure, digestate, toxinotypes, antimicrobial susceptibility

## Abstract

Digestate produced by agricultural biogas plants (BGPs) may contain pathogenic bacteria. Among them, *Clostridium perfringens* deserves particular attention due to its ability to grow under anaerobic conditions and persist in amended soil. The aim of this study was to examine the potential pathogenicity and the antimicrobial resistance of *C. perfringens* in manure and digestate collected from three agricultural biogas plants (BGPs). A total of 157 isolates (92 from manure, 65 from digestate) were screened for genes encoding seven toxins (*cpa*, *cpb*, *etx*, *iap* *cpe*, *netB*, and *cpb*2). The 138 *cpa* positive isolates were then screened for *tetA*(P), *tetB*(P), *tet*(M), and *erm*(Q) genes and tested for antimicrobial susceptibility. The toxinotypes identified in both manure and digestate were type A (78.3% of the isolates), type G (16.7%), type C (3.6%), and type D (1.4%), whereas none of the isolates were type F. Moreover, half of the isolates carried the *cpb2* gene. The overall prevalence of *tetA*(P) gene alone, *tetA*(P)-*tetB*(P) genes, and *erm*(Q) gene was 31.9, 34.8, and 6.5%, respectively. None of the isolates harbored the *tet*(M) gene. Multiple antimicrobial resistant isolates were found in samples that were collected from all the manure and digestates. Among them, 12.3% were highly resistant to some of the antibiotics tested, especially to clindamycin (MIC ≥ 16 µg/mL) and tilmicosin (MIC > 64 µg/mL). Some isolates were highly resistant to antibiotics used in human medicine, including vancomycin (MIC > 8 µg/mL) and imipenem (MIC > 64 µg/mL). These results suggest that digestate may be a carrier of the virulent and multidrug resistant *C. perfringens*.

## 1. Introduction

The number of biogas plants has increased considerably in Europe in the last decade (from 6227 installations in 2009 to 18,202 in 2018) [1], most of them operating with agricultural substrates. On-farm anaerobic digestion converts livestock manure into biogas and digestate, and the latter is commonly applied to agricultural soils. However, the spread of zoonotic pathogens and antibiotic resistant bacteria that are potentially present in digestate raises serious concerns. Among these bacteria, *Clostridium perfringens,* which causes food poisoning in humans and enteric diseases in domestic animals [2], deserves particular attention due to its persistence in soil amended with manure, digestate, or manure compost [3,4,5]. *Clostridia* are also suspected of playing a role in the dissemination of antibiotic resistance genes in manure-amended soil [4,5,6]. Moreover, *C. perfringens* has been suggested to be a reservoir for conjugative antimicrobial resistance genes [7,8].

The land application of manure may result in continuous exposure of the agricultural environment to pathogenic and antibiotic resistant bacteria [5]. This raises issues of human health and food safety [9]. Indeed, once contaminated livestock effluents are applied, direct transfer of pathogens to crops and animals may occur [10]. To date, there is no evidence of environmental transmission of *C. perfringens*, whether or not the bacterium is resistant to antibiotics but one possible transmission route to humans or animals is through the consumption of vegetables contaminated by livestock manure [11,12]. Whether anaerobic digestion of manure can reduce the environmental exposure to antimicrobial-resistant *C. perfringens* shed by farm animals has to be dully addressed. Given the increasing number of reports of antimicrobial resistance in anaerobes over the past two decades [13], it is important to examine the potential pathogenicity and antimicrobial profiles of *C. perfringens* present in digested manure before its application on agricultural land.

*C. perfrin*gens is a Gram-positive anaerobic spore forming bacterium that is responsible for various diseases, including food poisoning, gas gangrene, enteritis necroticans, and enterotoxemia [14]. It is also involved in antibiotic-associated diarrhea [15]. This bacterium is estimated to be the third most common bacterial cause of food-poisoning cases in the United States [16], and is the third most common cause of foodborne outbreaks in France [17]. *C. perfringens* produces more than 20 toxins and hydrolytic enzymes [18,19]. The species is divided into seven types, A to G, according to the presence and combination of four major toxins (α, β, ε and ι) encoded by genes *cpa*, *cpb*, *etx,* and *iap*, and two minor toxins (enterotoxin CPE and NetB) encoded by genes *cpe* and *netB* [15,19]. It also produces the β2 toxin encoded by the *cpb2* gene, which is implicated in several animal gastrointestinal diseases [20] and it is found in different *C. perfringens* toxinotypes [18]. 

Besides carrying toxin genes, the plasmids harbored by *C. perfringen*s also carry genes conferring resistance to antibiotics. The resistance of *C. perfringens* isolates to tetracycline, clindamycin, lincomycin, and erythromycin has been reported in animals [21,22]. Tetracycline resistance plasmids are the most common antibiotic resistance plasmids in *C. perfringens* [23]. The genes *tetA*(P) and *tetB*(P) are frequently associated with tetracycline resistance [24,25] and the genes *erm*(B) or *erm*(Q) have been associated with macrolide-lincosamide-streptogramin B resistance [23,26], the *erm*(Q) gene being the most common erythromycin resistance determinant reported in *C. perfringens*. Only a few studies have examined the antimicrobial susceptibility of *C. perfringens* isolates that were collected from agricultural environments. High resistance to tetracycline has been reported in isolates from piglets, chickens, or cattle [24,27,28,29]. Moreover, 63% and 38.4% of *C. perfringens* isolates collected from soil in the United States [28] and in Greece [30] were resistant to tetracycline. Soge et al. [8] also reported that the *tetA*(P) gene was common in isolates in water and soil. Despite the ability of *C. perfringens* to transfer antibiotic resistance via bacterial conjugation [7] and its ability to survive under conditions found in mesophilic anaerobic digesters (MAD) [31,32], information is lacking on the antimicrobial susceptibility of resistant *C. perfringens* isolates in digestate intended for land application. 

The aims of this study were (i) to identify the toxinotype and the antimicrobial resistance profiles of *C. perfringens* isolates in digestates originating from three agricultural biogas plants and (ii) to compare the profiles with *C. perfringens* isolates in manure that was collected in the same biogas plants. 

## 2. Materials and Methods 

### 2.1. Biogas Plants and Sample Collection

The samples were collected from three on-farm biogas plants (BGPs) located in a same area (within a distance of less than 120 km), fed with manure and co-substrates of vegetable origin, the proportion of which depending on the BGP (Table S1). BGP1 and BGP3 were fed with liquid pig manure. BGP2 manure mainly comprised liquid dairy manure (≥86%) and to a lesser extent, solid poultry manure. 

Each BGP was sampled eight times (at six-week intervals) from May 2017 to July 2018. Prior to sampling, the liquid manure in the storage tank and in the digester was mixed. At each sampling date, liquid manure and raw digestates were collected using three 10 L buckets for each type of matrix. Samples from each bucket were transferred into 1 L sterile flasks. A total of 48 samples per BPG were analyzed (2 matrices × 3 replicates × 8 sampling dates). Moreover, as poultry manure was not systematically present in BGP2, only three samples were collected during the sampling period. Microbiological analyses were performed within 4 h after sampling. 

### 2.2. Isolation of C. perfringens 

Samples (25 g) of manure or digestate were homogenized in 225 mL of peptone water. Tenfold serial dilution of the suspensions was performed. *C. perfringens* was isolated according to the standard ISO method 7937:2005-02 [33]. Briefly, Trytone sulfite Cycloresrin agar (TSC, Thermo Fisher Scientific, Courtaboeuf, France) was poured into a Petri dish containing 1 mL of each serial dilution. After the solidification of the agar, an overlayer of 10 mL of TSC was added. The plates were incubated under anaerobic conditions while using anaerobic jars with gaspack (AnaeroGen, Thermo Scientific, Courtaboeuf, France) at 37 °C for 20 h ± 2 h. Five black colonies were randomly selected on a plate containing between five and 10 colonies of presumptive *C. perfringens* and transferred into thioglycolate broth (Thermo Fisher Scientific, Courtaboeuf, France). The tubes were incubated under anaerobic conditions at 37 °C for 18 h ± 2 h. Five drops of each thioglycolate culture were transferred into Lactose Sulfite Broth (Biokar Diagnostics, Allonne, France). After incubation at 46 °C for 18 h ± 2 h in a water bath, the tubes exhibiting gas production and blackening of the culture medium were considered positive. At each sampling date, all of the confirmed colonies were purified and suspended in 50% glycerol/50% Brain Heart Infusion Broth (BHI, Thermo Fisher Scientific, Courtaboeuf, France) before being stored at −80 °C. A total of 157 isolates were collected from the three BGPs (Table 1). 

### 2.3. DNA Extraction

All of the isolates were cultured on Wilkins-Chalgren agar (WICH, Thermo Fisher Scientific, Courtaboeuf, France). After incubation at 37 °C for 24 h under anaerobic conditions, ca. 40 mg of pure culture were removed with a sterile swab and transferred into a 2 mL tube. DNA extractions were performed using the Nucleospin^®^ Microbial DNA kit (Macherey-Nagel, Hoerdt, France) according to the supplier’s recommendations. The DNA was eluted from the silica column in a 100 μL elution buffer (5 mM Tris/HCl, pH 8.5) and then stored at −20 °C. DNA purity and concentration were determined by measuring absorption at 260 and 280 nm while using a BioPhotometer Spectrophotometer (Eppendorf, Montesson, France). 

### 2.4. Determination of the Toxin Genotype and Search for Antibiotic Resistance Genes

Real-time PCR targeting the four major toxin genes (*cpa*, *cpb*, *etx*, and *iap),* and three minor toxin genes (*cpe*, *netB*, and *cpb2)* was carried out to genotype the *C. perfringens* isolates (Table 2). Strain 103,009,089 from the ANSES (French Agency for Food, Environmental and Occupational Health and Safety) strain collection and strains CIP 103409^T^, CIP 106527, CIP 105568, CIP 106156, and CIP 104612 from the Institut Pasteur Collection were used as positive controls. 

Four antibiotic resistance genes that may be carried by *C. perfringens* (*tetA*(P), *tetB*(P), *tet*(M), and *erm*(Q)) were detected by real-time PCR (Table 2)**.** A strain of *C. perfringens* and a strain of *Clostridioides difficile*, kindly provided by Dr. O. Firmesse (Anses, Maisons-Alfort, France) and Dr. F. Barbut (Saint-Antoine Hospital, Paris, France), respectively, were used as positive controls for *tetA*(P), *tetB*(P), and *erm*(Q) genes *(C. perfringens* 175 BCL 67*)*, and for *tet*(M) *(C. difficile* CD17_412). 

Real-time PCRs were performed using a Bio-Rad CFX96 real-time PCR machine with Bio-Rad CFX Manager software, version 1.1 (Bio-Rad, Marnes-la-coquette, France) in a final volume of 25 µL. The reaction mixture consisted of 12.5 µL of IQ SYBR Green Supermix (Bio-Rad, Marnes-la-coquette, France), a 400 nmol/L concentration of each primer, 2 µL of 1/10 diluted DNA, and 9.5 µL of water. Negative controls without DNA were also prepared. PCR preparation was performed while using the epMotion 5070 automated pipetting station (Eppendorf, Montesson, France). 

The Real-time PCR program for the target gene included one cycle at 94 °C for 5 min, 40 cycles at 94 °C for 20 s, one cycle at 55 °C for 30 s, and one cycle at 72 °C for 30 s [34,35]. The melting curve of the amplification products was analyzed at the end of each qPCR (temperature range 65 °C to 95 °C). Analysis of the melting curve of the standard replicates only yielded one peak resulting from the specific amplification of the target gene. For a given gene, the amplification of the correct PCR fragment was verified by comparing the cycling threshold value and the melting curve of the standard PCR product with each environmental isolate PCR product. 

### 2.5. Testing Antimicrobial Susceptibility 

Antimicrobial susceptibility was tested on the 138 *cpa*-positive isolates using the commercially available Sensititre™ Bovine/porcine according to the manufacturers’ instructions (ThermoFisher Diagnostics, Courtaboeuf, France). Seventeen antimicrobial agents commonly used for the treatment of livestock diseases were tested. They included (µg/mL) penicillin (PEN, 0.12–8), ampicillin (AMP, 0.25–16), ceftiofur (TIO, 0.25–8), tartrate tylosin (TYLT, 0.5–32), tulathromycin (TUL, 1–32), tilmicosin (TIL, 4–64), tiamulin (TIA, 0.5–32), chlortetracycline (CTET, 0.5–8), oxytetracycline (OXY, 0.5–8), danofloxacin (DANO, 0.125–1), enrofloxacin (ENRO, 0.125–2), trimethoprim/sulfamethoxazole (SXT, 2/38), sulfadimethoxine (SDM, 256), neomycin (NEO, 4–32) florfenicol (FFN, 0.25–8), spectinomycin (SPE, 8–64), and clindamycin (CLI, 0.25–16). The plates contained three wells not inoculated with antibiotics as growth controls. The microplates were sealed with a micro-perforated film and incubated at 37 °C for 24 h under anaerobic conditions. 

The isolates were incubated in WICH broth at 37 °C in under anaerobic conditions. Overnight bacterial cultures were adjusted to 0.5 McFarland turbidity standard in WICH broth, and 50 µL of the diluted bacterial suspensions (*ca.* 10^5^ CFU/well) were added to each well. 

Among the 138 *cpa*-positive isolates, 14 isolates originated from the three BGPs were selected for their high resistance to antibiotics of the Sensititre™ Bovine/porcine plate. Their susceptibility to antimicrobial agents commonly used against anaerobic human pathogens were tested with the Sensititre™ Anaerobe MIC plate (WICH, Thermo Fisher Scientific, Courtaboeuf, France) according to the manufacturers’ instructions (ThermoFisher Diagnostics, Dardilly, France). The plate contained 14 antimicrobial agents (µg/mL): metronidazole (MRD, 0.5–32), linezolid (LZD, 1–16), moxifloxacin (MXF, 0.12–4), tigecycline (TGC, 1–32), piperacillin-tazobactam constant 4 (P/T4, 2/4–64/4), piperacillin (PIP, 2–64), penicillin (PEN, 0.06–8), vancomycin (VAN, 1–8), amoxicillin (AMOX, 0.25–32) chloramphenicol (CHL, 2–16), amoxicillin/clavulanic acid 2:1 ratio (AUG2, 0.25/0.12–32/16), clindamycin (CLI, 0.5–64), rifampicin (RIF, 1–64), and imipenem (IMI, 0.12–64). The microplates were sealed with a micro-perforated film and incubated at 37 °C for 48 h under anaerobic conditions. The reference strain CIP103409 ^T^ (Institut Pasteur Collection, Paris, France) was used as control. The antimicrobial profiles that were obtained on the two Sensititre™ plates are presented in Tables S2 and S3.

The lowest concentration at which no bacterial growth was detected corresponded to the minimum inhibitory concentration (MIC) value. MIC 50 and MIC 90 were the MIC values at which 50% and 90% of the isolates were inhibited. 

### 2.6. Statistical Analysis

The MIC of the isolates for the tested antimicrobials was subjected to principal components analysis (PCA) to explore the relationship between the origin of the isolate (BGP, manure, digestate) and their antimicrobial resistance profiles. All of the statistical tests were performed with XLSTAT 2019 (Addinsoft, Bordeaux, France). Heatmap of normalized antimicrobial resistance were generated using the Complex Heat map package in Circlize in R [36]. 

## 3. Results

### 3.1. Toxinotyping of C. perfringens Isolates

Among the 157 isolates, 138 (88%) carried the *cpa* gene (43 from BGP1, 47 from BGP2, and 48 from BGP3). As shown in Table 3, 108 (78.3%) were type A, and 23 (16.7%) were type G (*netB* gene). Both types were found in pig and dairy manure and in the digestate in all three BGPs. Five isolates (3.6%) and two isolates (1.4%), found only in BGP3, were type C (*cpb* gene) and type D (*etx* gene), respectively. The proportion of *C. perfringens* isolates harboring the *cpb2* gene were 48.1%, 50%, 56.5%, and 80% for types A, D, G, and C, respectively. None of the isolates harbored both *cpe* and *iap* genes.

### 3.2. Tetracycline and Erythromycin Resistance Genes of C. perfringens Isolates

Both *tetA*(P) and *tetB*(P) genes were detected in the 138 *cpa*-positives isolates from manure and digestate of the three BGPs, but not in isolates from poultry manure (Table 4), whereas the *erm*(Q) gene was only found in isolates from BGP1 and BGP3. 

The most frequent gene, *tetA*(P)*,* was found in 62.5% and 86.2% of the isolates from the manure and digestate in the three BGPs, respectively. The *tetB*(P) gene was associated with *tetA*(P) genes in 31.3% and 50% of the isolates from manure and digestate, respectively. The *erm*(Q) gene was found less frequently, i.e., in 3.8% of the isolates from manure and 10.3% of the isolates from digestate. No *tet*(M) gene was detected in the isolates. 

### 3.3. Antimicrobial Susceptibility of C. perfringens Isolates

**Figure 1:**
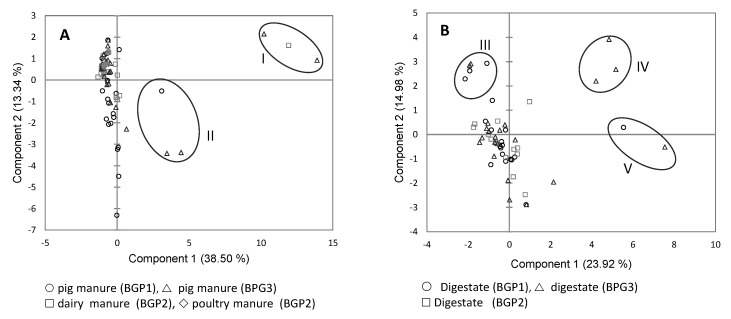
Principal components analysis (PCA) of antimicrobial resistance profiles of *C. perfringens* isolates from manure (**A**) and digestate (**B**) in three BGPs. Groups I to V contained highly resistant isolates.

The 138 *cpa*-positive isolates were subjected to antimicrobial resistance analysis while using the Sensititre^TM^ bovine/porcine test system. Plots of the first and second PCA scores revealed no differences between the antimicrobial resistance profiles of the isolates originating from the manure (Figure 1A) and from the digestates (Figure 1B) of the three BGPs. However, 17 isolates originating from the three BGPs, which we clustered in five groups (I and II, Figure 1A; III to V, Figure 1B), differed from the other isolates by their high resistance to some of the antibiotics tested (Figure S1). Isolates from Group I (*n* = 3) differed by their high level of resistance to penicillin (MIC > 8 µg/mL), ampicillin and clindamycin (MIC ≥ 16 µg/mL), tartrate tylosin and tiamulin (MIC > 32 µg/mL), and tilmicosin (MIC > 64 µg/mL). Isolates from Group II (*n* = 3) were highly resistant to clindamycin (MIC ≥ 16 µg/mL), tilmicosin (MIC > 64 µg/mL), and oxytetracycline (MIC > 8 µg/mL). Isolates from Group III (*n* = 6) had a higher MIC for enrofloxacin and danofloxacin than the maximum concentration tested (MIC > 2 µg/mL and > 1 µg/mL, respectively). Isolates from Group IV (*n* = 3) were highly resistant to both clindamycin (MIC ≥ 16 µg/mL) and tilmicosin (MIC > 64 µg/mL). Isolates from Group V (*n* = 2) were highly resistant to oxytetracycline (MIC > 8 µg/mL), clindamycin (MIC > 16 µg/mL), and tilmicosin (>64 µg/mL). 

The antimicrobial resistance profiles of the isolates varied considerably in the biogas plants, regardless of their origin (manure or digestate) (Figure S1). However, the MIC distribution of isolates from manure was close to that of isolates from digestate (Table 5, Figure S2). 

With the exception of tulathromycin, the MICs of the isolates covered the dilution range tested for all antimicrobials (Table 5). Most of the isolates were susceptible to β-lactam and macrolides, except tulathromycin (with a MIC 50 value of 64 µg/mL). Enrofloxacin and danofloxacin had low MIC 50 values of 0.25–0.5 and 1 µg/mL, respectively. Most of the isolates exhibited sensitivity to chlortetracycline with a MIC 50 value of ≤0.50 µg/mL in manure and of 1 µg/mL in digestate. The MIC 50 values of oxytetracycline were higher (1 µg/mL in manure and 8 µg/mL in digestate).

Moreover, the growth of 19% of *C. perfringens* isolates was inhibited by sulfadimethoxine (Figure S1). Among the highly resistant group I to V, 14 isolates were tested on microplates that contained antibiotics used in human medicine (Table S3). Five of the tested isolates present in BGP2 and BGP3, showed high resistance to human antibiotics including imipenem, linezolid, amoxicillin-clavulanate, chloramphenicol, moxifloxacin, tigecycline, and vancomycin.

## 4. Discussion

Although digestates from biogas plants fed with manure contain high levels of *C. perfringens* (*ca.* 10^4^ CFU/g) [32,33,34,35,36,37], no information is available on the range of toxin genes and the antimicrobial profiles of this pathogenic bacterium in agricultural digestate that is intended for land application. This is the first study to assess digestate as a potential carrier of virulent and multiresistant *C. perfringens*. 

### 4.1. Toxinotyping of C. perfringens Isolates

The dominance of type A isolates in both manure and digestate is in agreement with the high prevalence of this toxinotype in farm animals [38,39,40]. Although the *cpe* gene was not detected in either manure or digestate, the three BGPs produced digestate containing virulent *C. perfringens.* Indeed, half the type A isolates from the three BGPs carried the *cpb2* gene encoding the β2 toxin, which is reported to be associated with diarrheal diseases [19,41]. This result is consistent with those of studies that were conducted on farm animals, in which between 39% and 78.8% frequency of the *cpa* gene together with *cpb2* gene was observed [25,42,43,44]. Moreover, some isolates recovered from manure and digestate were positive for *cpb*, *etx*, and *netB* genes. Conversely, Chan et al. [45] did not detect these genes in fecal and manure samples collected from pig farms. Type C (*cpa* + *cpb* genes) and type D (*cpa* + *etx* genes), both associated with farm animal diseases, were detected at a low frequencies only in isolates from the BGP3 digestate. Type G (*cpa* and *netB* genes), responsible for necrotic enteritis in chickens [6,29,45], was carried by isolates from all three BGPs, showing that land application of digestate can contribute to the dissemination of *C. perfringens* carrying *netB* in the agricultural environment.

### 4.2. Tetracycline and Erythromycin Resistance Genes of C. perfringens Isolates

The occurrence of resistance genes and the antimicrobial susceptibility of *C. perfringens* of environmental origin have rarely been studied. In this study, although the *tet*(M) gene was not detected in digestate, the *tetA*(P) gene alone and double resistance *tetA*(P)*-tetB*(P) genes were found in 34.5% and 41.4% of the isolates, respectively. Their prevalence in digestates was slightly higher than that reported in other matrices. Indeed, Kiu et al. [14] observed a frequency of detection of *tetA*(P) alone and of the *tetA*(P)*-tetB*(P) gene of 14.8% and 35.2% in isolates from broiler chickens, respectively. In another study, they were detected in 26% and 22% of isolates of environmental origin (soils, water, and sewage) [8]. The proportion of isolates carrying the *erm*(Q) gene (13.6%) in the digestates of the two BGPs fed with pig manure was also higher than that previously observed in isolates that were collected from broiler chickens (4.5%) and in environmental samples (1%) [8,14]. 

### 4.3. Antimicrobial Susceptibility of C. perfringens Isolates

In addition to their natural resistance to aminoglycosides*, C. perfringens* showed significant resistance to tetracycline, which has been associated with the presence of *tetA*(P), *tetB*(P), and *tet*(M) genes [27]. In this study, the isolates were more resistant to oxytetracycline than to chlortetracycline (Table 5), in agreement with the studies of Martel et al. [46] and Ngamwongsatit et al. [38] who reported that chlortetracycline was more active than oxytetracycline against *C. perfringens* isolated from chickens and from neonatal piglets, respectively. It is noteworthy that 7.4% of the isolates with a MIC ≥ 4 µg/mL for chlortetracycline and with a MIC ≥ 8 µg/mL for oxytetracycline harbored none of the three target genes (*tetA*(P), *tetB*(P), and *tet*(M)). This could be explained by the presence of other tetracycline resistance genes. Indeed, *tet*(K) and *tet*(L) have been detected at low frequencies in isolates from broilers [46,47]. Moreover, 4.4% of the isolates that were susceptible to both chlortetracycline and oxytetracycline (MIC ≤ 0.5 µg/mL) carried the *tetA*(P)*-tetB*(P) genes and 22.2% carried the *tetA*(P) gene. The inconsistency between the MIC values and the presence of genetic tetracycline resistance determinants has already been pointed out [7,24,47]. The reason for the presence of sensitive isolates harboring *tetA*(P) and/or *tetB*(P) is unclear. This may be due to lack of expression or to point mutation, as suggested by Kather et al. [7] and Gholamiandehkordi et al. [47]. In the present study, all strains were highly resistant to tulathromycin, a macrolide antibiotic used to treat bovine and swine respiratory diseases [48]. However, it was not possible to determine the antibiotic use in the three farms whose manure was treated by anaerobic digestion. On the whole, the MIC 50 observed in manure and digestates for the antibiotic tested resembled those reported in animal feces [22,27,29,38,49,50], suggesting that storing manure and anaerobic digestion have no impact on the antimicrobial susceptibility of *C. perfringens* originating from farm animals. 

Among the *C. perfringens* isolates from the three BGPs, 12.3% were highly resistant to some of the antibiotics tested. Five of the 14 isolates tested with the Sensititre™ Anaerobe MIC plate (four from BGP3, one from BGP2) were also highly resistant to human antimicrobial medicine (Table S3). Based on the values of the EUCAST anaerobe MIC breakpoints reported by Pence et al. [13], these isolates were resistant to metronidazole, clindamycin, and penicillin (MIC ≥ 8 µg/mL), to chloramphenicol, amoxicillin-clavulanate, and imipenem (MIC ≥ 16 µg/mL), and to piperacillin-tazobactam (MIC > 64 µg/mL). High MIC were also observed for vancomycin (MIC > 8 µg/mL), which is normally low for clostridia, and for moxifloxacin (MIC > 4 µg/mL), the resistance to which is reported to be increasing [13]. 

In this study, digestates were collected from digesters fed with manure, which is the first step of the process of anaerobic digestion. Indeed, post-treatments of raw digestates involved either storage in a tank, post-digestion (BGP2), or mechanical separation of the solid fraction before composting and storage of the liquid fraction (BGP1). It has been reported that post-digestion and mechanical separation do not reduce the concentration of *C. perfringens* [37], contrary to manure composting [3,51]. Moreover, the legal requirement for heat treatment (70 °C for 1 h) according to the EU animal by-products regulation (EU Commission Regulation (EU) No 142/2011) is not sufficient for eliminating spores of *C. perfringens*. Indeed, Sahlström et al. [52] reported that the number of *C. perfringens* was not significantly affected by this time-temperature condition. On the opposite, high temperatures (135 °C to 190 °C) pre-treatments performed in order to increase accessibility of the enzymes to the substrate and, therefore, to enhance biogas production [53,54], may reduce the concentration of *C. perfringens*. Actually, the inactivation of *C. perfringens* spores can be achieved at temperatures of 110 °C [55]. Thus, high temperature pre-treatment or digestate composting may be applied to avoid *C. perfringens* spreading in soils.

## 5. Conclusions

The results of this study revealed that both manure and digestates contained *C. perfringens* isolates belonging types C, D, and G, which may be pathogenic for human or animals. Moreover, the presence in the digestates of some isolates highly resistant to antibiotics used in both veterinary and human medicine may increase the burden of antimicrobial resistance in agroecosystems, as *C. perfringens* could be involved in horizontal gene transfer from enteric bacteria to bacterial soil populations.

## Figures and Tables

**Table 1:** Distribution of the isolates collected from the three biogas plants (BGPs).

	BGP1		BGP2			BGP3	
Matrix	Manure	Digestate	Dairy Manure	Poultry Manure	Digestate	Manure	Digestate
Nb isolates	24	21	25	14	19	29	25

**Table 2:** Primers used for the detection of *C. perfringens* toxin genes and antimicrobial resistance genes.

Gene	Primers	Sequences (5′-3′)	Amplicon Size (bp)	Reference
*cpa*	CPA5L	AGTCTACGCTTGGGATGGAA	900	[35]
	CPA5R	TTTCCTGGGTTGTCCATTTC		
*cpb*	CPBL	TCCTTTCTTGAGGGAGGATAAA	611	[35]
	CPBR	TGAACCTCCTATTTTGTATCCCA		
*iap*	CPIL	AAACGCATTAAAGCTCACACC	293	[35]
	CPIR	CTGCATAACCTGGAATGGCT		
*etx*	CPETXL	TGGGAACTTCGATACAAGCA	396	[35]
	CPEXTR	TTAACTCATCTCCCATAACTGCAC		
*cpe*	CPEL	GGGGAACCCTCAGTAGTTTCA	506	[35]
	CPER	ACCAGCTGGATTTGAGTTTAATG		
*cpb2*	CPB2L	CAAGCAATTGGGGGAGTTTA	200	[35]
	CPB2R	GCAGAATCAGGATTTTGACCA		
*netB*	AKP78	GCTGGTGCTGGAATAAATGC	383	[34]
	AKP79	TCGCCATTGAGTAGTTTCCC		
*erm*(Q)	GE 350	GAAGAGTTAAATYCACCAACTGA	84	This study
	GE 352	ACTCTCTCTAGGTATTCCCA		
*tetA*(P)	GE 353	TGTAGCACAGATTGTATGGGGA	124	This study
	GE 355	CCCTGCTTGTGCTCCCTTTA		
*tetB*(P)	GE 359	TTTTGGGCGACAGTAGGCTT	90	This study
	GE 361	TGGCAATGACCCTACTGAAACA		
*tet*(M)	GE 362	GCTATTGCCACAGAGAGAGAGA	127	This study
	GE 364	CGGGTCACTGTCGGAGATTT		

**Table 3:** Occurrence of the toxinotypes of *C. perfringens* isolates in manure and digestate in three biogas plants.

Type (Gene)	BGP1 *n* (%)		BGP2 *n* (%)			BGP3 *n* (%)		Total *n* (%)	
	Manure *n* = 23	Digestate *n* = 20	Dairy Manure *n* = 19	Poultry Manure *n* = 14	Digestate *n* = 14	Manure *n* = 24	Digestate *n* = 24	Manure *n* = 80	Digestate *n* = 58
A (*cpa*)	6 (26.1)	3 (15)	15 (78.9)	12 (85.7)	8 (57.1)	6 (25)	6 (25)	39 (67.2)	17(21.3)
A (*cpa + cpb2*)	13 (56.5)	15 (75)	2(10.5)	2(14.3)	5 (35.7)	8 (33.3)	7 (29.2)	25 (43.1)	27(33.8)
G (*netB)*	2 (8.7)	1 (5)	2(10.5)	-	-	4 (16.7)	1 (4.2)	8 (13.8)	2(2.5)
G (*netB + cpb2)*	2 (8.7)	1 (5)	-	-	1 (7.1)	3 (12.5)	6 (25)	5 (8.6)	8(10)
C (*cpb*)	-^1^	-	-	-	-	-	1 (4.2)	-	1 (1.3)
C (*cpb + cpb2*)	-	-	-	-	-	1 (4.2)	3 (12.5)	1 (1.7)	3(3.8)
D (*etx*)	-	-	-	-	-	1 (4.2)	-	1 (1.7)	-
D (*etx + cpb2*)	-	-	-	-	-	1 (4.2)	-	1 (1.7)	-

^1^ not detected.

**Table 4:** Detection of antimicrobial resistance genes (*tetA*(P), *tetB*(P), *tet*(M), and *erm*(Q) genes) of isolates in manure and digestate in three biogas plants.

Gene	BGP1 *n* (%)		BGP2 *n* (%)			BGP3 *n* (%)		Total *n* (%)	
	Manure *n* = 23	Digestate *n* = 20	Dairy Manure *n* = 19	Poultry Manure *n* = 14	Digestate *n* = 14	Manure *n* = 24	Digestate *n* = 24	Manure *n* = 80	Digestate *n* = 58
*tetA*(P)	12 (52.2)	13 (65)	2(10.5)	-	2 (14.3)	10 (41.7)	5 (20.8)	24 (30)	20 (34.5)
*tetA*(P) *+ tetB*(P)	9 (39.2)	6 (30)	10 (52.6)	-	8 (57.1)	5 (20.8)	10 (41.7)	24 (30)	24 (41.4)
*tetA*(P) *+ erm*(Q)	1 (4.3)	-	-	-	-	-	1 (4.2)	1 (1.25)	1 (1.7)
*tetB*(P) *+ erm*(Q)	- ^1^	-	-	-	-	1 (4.2)	-	1 (1.25)	-
*tetA*(P) *+ tetB*(P) *+ erm*(Q)	1 (4.3)	1 (5)	-	-	-	-	4 (16.7)	1 (1.25)	5 (8.6)
*tet*(M)	-	-	-	-	-	-	-	-	-
no ARG *^2^*	-	-	7 (36.8)	14 (100)	4 (28.6)	8 (33.3)	4 (16.7)	29 (36.3)	8 (13.8)

^1^ not detected; ^2^ no detection of the four genes.

**Table 5:** Minimum inhibitory concentration (MIC) distribution (µg/mL) of 13 antimicrobials tested against 138 isolates of *C. perfringens* originating from three BPGs.

	Antimicrobial Agents	Origin of Isolates							
		Manure (*n* = 80)				Digestate (*n* = 58)			
		MIC50	MIC90	Min	Max	MIC50	MIC90	Min	Max
β-lactams	Penicillin	≤0.125	1	≤0.125	>8	≤0.125	0.5	≤0.125	1
	Ampicillin	≤0.25	≤0.25	≤0.25	>16	≤0.25	≤0.25	≤0.25	2
	Ceftiofur	≤0.25	1	≤0.25	4	≤0.25	2	≤0.25	2
Macrolides	Tartrate Tylosin	≤0.5	4	≤0.5	>32	≤0.5	8	≤0.5	16
	Tilmicosin	≤4	8	≤4	>64	≤4	≤4	≤4	>64
	Tulathromycin	64	>64	16	>64	64	>64	32	>64
Lincosamides	Clindamycin	2	2	≤0.25	>16	2	4	≤0.25	>16
Fluoroquinolones	Enrofloxacin	0.25	>2	0.25	>2	0.50	>2	≤0.125	>2
	Danofloxacin	1	>1	0.25	>1	1	>1	0.5	>1
Pleuromutilins	Tiamulin	4	8	≤0.5	>32	4	16	≤0.5	>32
Phenicols	Florfenicol	1	2	0.5	>8	1	1	≤0.25	2
Tetracyclines	Chlortetracycline	≤0.5	8	≤0.5	>8	1	4	≤0.5	8
	Oxytetracycline	2	>8	≤0.5	>8	8	>8	≤0.5	>8

## Supplementary Materials

The following are available online at https://www.mdpi.com/1660-4601/17/15/5450/s1, Figure S1: Heatmap of antimicrobial resistance profiles of *C. perfringens* isolates from three biogas plants, Figure S2: MIC distribution of 13 antimicrobials in manure and raw digestate in three BGPs, Table S1: Process parameters of the three biogas plants, Table S2: MIC of *C. perfringens* CIP 103409^T^ obtained on Sensititre^TM^ Bovine/porcine (µg/mL), Table S3: MIC of selection of isolates (belonging to groups I to V) from the three BGP and of the reference strain CIP 103409^T^ obtained on Sentititre^TM^ FRAM1ANA (µg/mL).
