# Supplementary material for: Characterization of Clostridium Perfringens Isolates Collected from Three Agricultural Biogas Plants over a One-Year Period
Source: Int J Environ Res Public Health. 2020 Jul 29;17(15):5450. doi: 10.3390/ijerph17155450 (PMC7432756; doi:10.3390/ijerph17155450)
Supplement: Supplementary file 1 [file ijerph-17-05450-s001.zip › Supplementary data FigureS1 23 07.pdf]

Supplementary data

Characterization of *Clostridium perfringens* isolates collected from three agricultural biogas plants over a one year period.

Lorine Derongs, Céline Druilhe, Christine Ziebal, Caroline Le Maréchal, Anne-Marie Pourcher

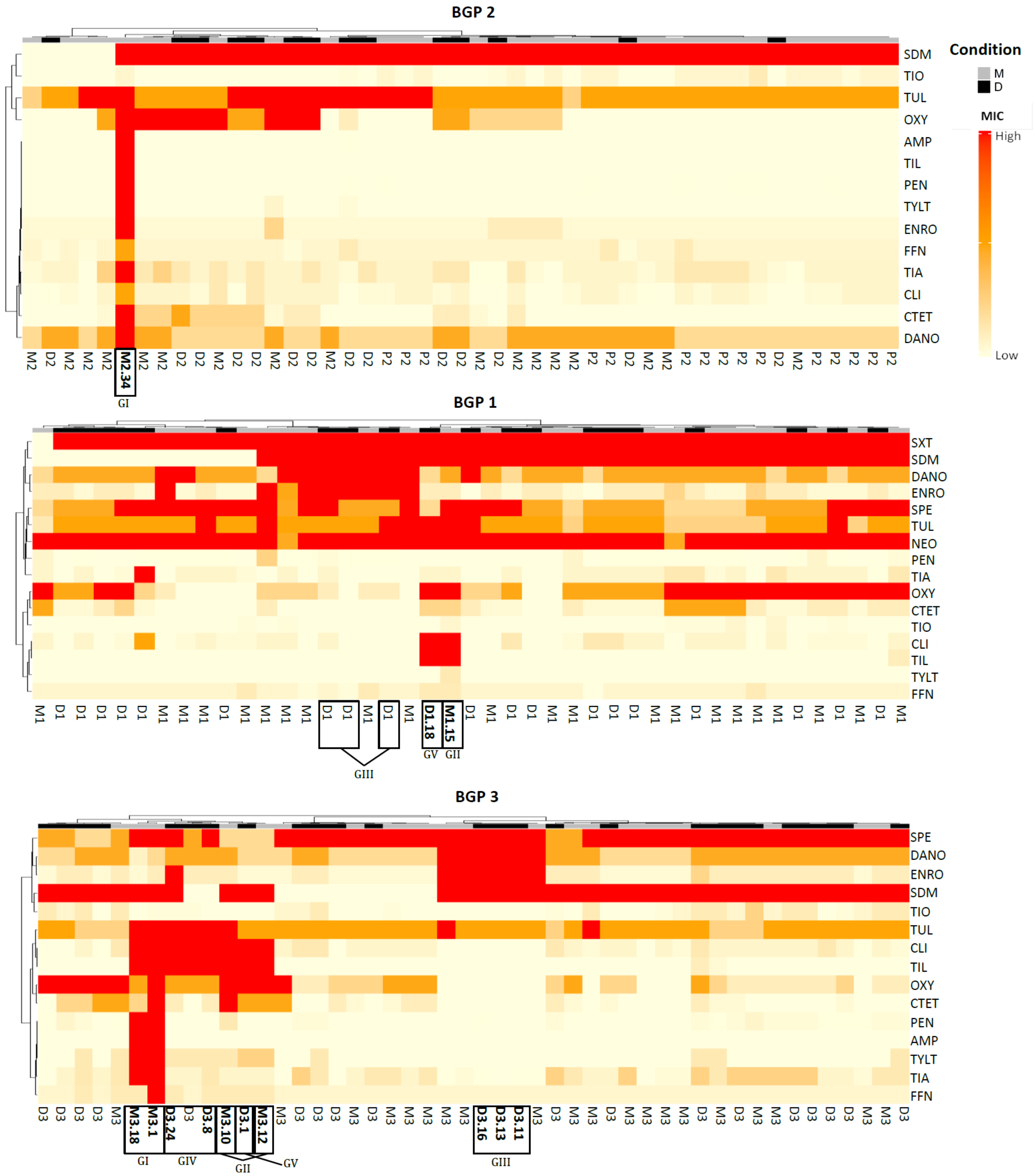

Figure S1 : Heatmap of antimicrobial resistance profiles of *C. perfringens* isolates from three biogas plants (BGP1, BGP2, BGP3). Isolates from pig and dairy manure (M), poultry manure (P) and raw digestates (D). The numbered isolates correspond to isolates that were highly resistant to the antibiotics tested. GI to GV correspond to the groups shown in Figure 2. PEN: penicillin, AMP: ampicillin, TIO : ceftiofur, TYLT: tartrate tylosin, TUL: tulathromycin, TIL: tilmicosin, TIA: tiamulin, CTET: chlortetracycline, OXY: oxytetracycline, DANO: danofloxacin, ENRO: enrofloxacin, SXT: trimethoprim / sulfamethoxazole, SDM sulfadimethoxine, NEO: neomycin FFN: florfenicol, SPE: spectinomycin, CLI: clindamycin.
