# Supplementary material for: Characterization of Clostridium Perfringens Isolates Collected from Three Agricultural Biogas Plants over a One-Year Period
Source: Int J Environ Res Public Health. 2020 Jul 29;17(15):5450. doi: 10.3390/ijerph17155450 (PMC7432756; doi:10.3390/ijerph17155450)
Supplement: Supplementary file 1 [file ijerph-17-05450-s001.zip › Supplementary data FigureS2 23 07.pdf]

Supplementary data

Characterization of *Clostridium perfringens* isolates collected from three agricultural biogas plants over a one year period.

Lorine Derongs, Céline Druilhe, Christine Ziebal, Caroline Le Maréchal, Anne-Marie Pourcher

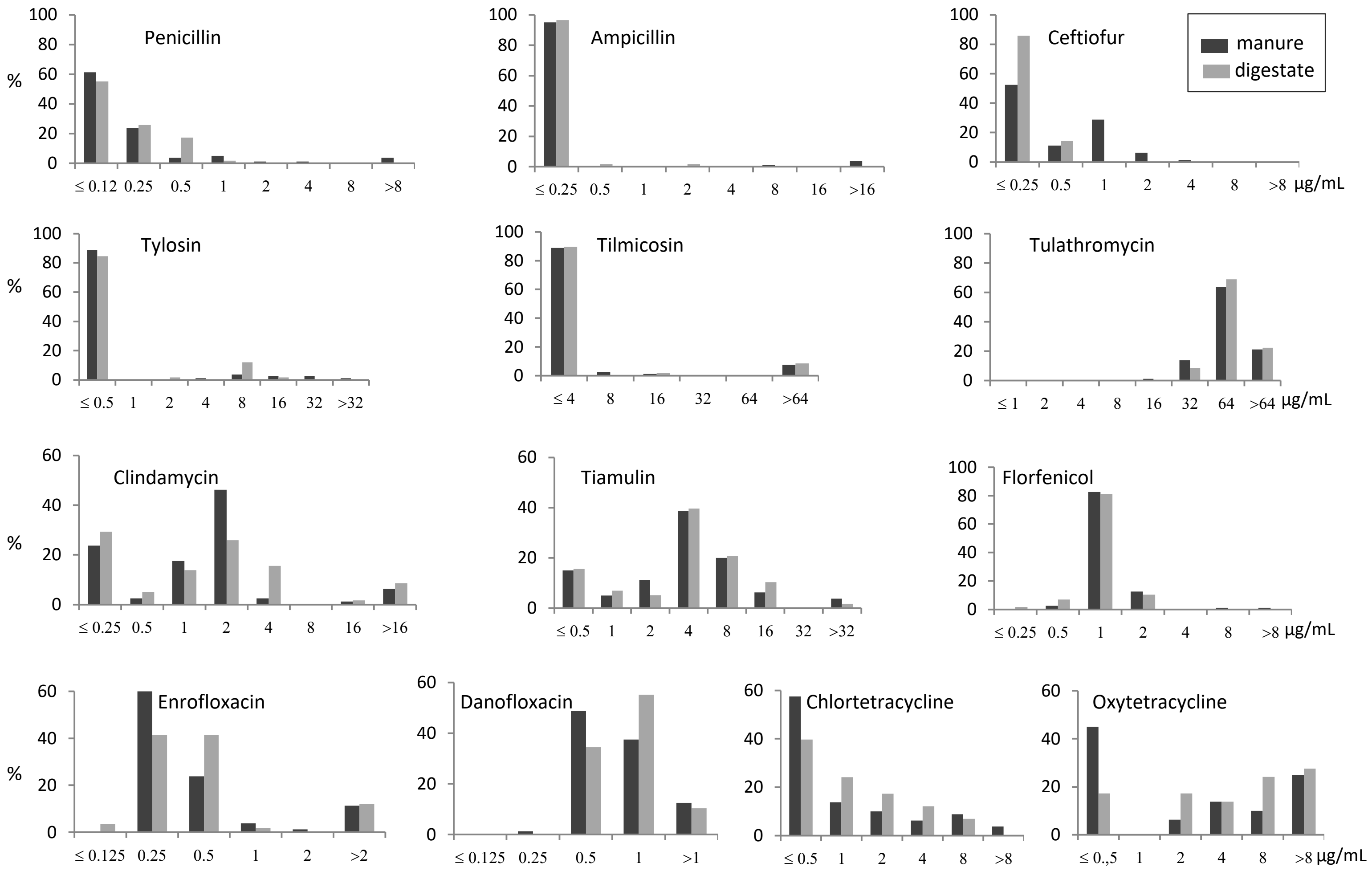

Figure S2. MIC distribution of 13 antimicrobials in manure and raw digestate in three BGPs.
