# Supplementary material for: Characterization of Clostridium Perfringens Isolates Collected from Three Agricultural Biogas Plants over a One-Year Period
Source: Int J Environ Res Public Health. 2020 Jul 29;17(15):5450. doi: 10.3390/ijerph17155450 (PMC7432756; doi:10.3390/ijerph17155450)
Supplement: Supplementary file 1 [file ijerph-17-05450-s001.zip › Supplementary Tables 220720 (3).pdf]

Supplementary data

**Characterization of *Clostridium perfringens* isolates collected from three agricultural biogas plants over a one-year period.**

Lorine Derongs, Céline Druilhe, Christine Ziebal, Caroline Le Maréchal, Anne-Marie Pourcher.

**Table S1. Process parameters of the three biogas plants.**

| Biogas plant | composition of the manure | Proportion of manure among feedstock (%) | Hydraulic retention time (day) | Average temperature (°C) |
|---|---|---|---|---|
| BGP1 | pig manure | 66.1 ± 6.4 | 42.7 ± 4.2 | 40.0 ± 0.2 |
| BGP2 | dairy and poultry manure [a] | 59.2± 5.6 | 73.1 ± 7.5 | 40.2 ± 0.9 |
| BGP3 | pig manure | 98 ± 1.8 | 42.3 ± 6.5 | 32.7 ± 5.2 |

[a] proportion of dairy manure / poultry manure of 7/1 (w/w)

**Table S2. MIC of *C. perfringens* CIP 103409$^T$ obtained on Sensititre$^{TM}$ Bovine/porcine (µg/mL).**

| strain | PEN[a] | AMP | TIO | TYLT | TIL | TUL | CLI | ENRO | DANO | TIA | FFN | CTET | OXY |
|---|---|---|---|---|---|---|---|---|---|---|---|---|---|
| **CIP 103409$^T$** | ≤0.125 | ≤0.25 | ≤0.25 | ≤0.5 | 8 | >64 | <0.25 | 0.25 | 0.5 | 1 | 1 | ≤0.5 | ≤0.5 |

[a]: PEN:Penicillin, AMP: Ampicillin, TIO: Ceftiofur, TYLT: Tartrate Tylosin, TIL: Tilmicosin, TUL: Tulathromycin, CLI: Clindamycin, ENRO : Enrofloxacin, DANO: Danofloxacin, TIA: Tiamulin, FFN: Florfenicol, CTET: Chloretracucline, OXY: Oxytetracycline

**Table S3. MIC of selection of isolates from the three BGP and of the reference strain CIP 103409[T] obtained on Sentititre FRAM1ANA (µg/mL)**

| Isolates [a] (Group) | MRD[b] | LZD | MXF | TGC | P/T4 | PIP | PEN | VAN | AMOX | CHL | AUG2 | CLI | RIF | IMI |
|---|---|---|---|---|---|---|---|---|---|---|---|---|---|---|
| **CIP 103409[T]** | >32 | 4 | 0.5 | ≤0.5 | ≤1/≤2 | ≤1 | 0.5 | ≤0.5 | ≤0.125 | 4 | ≤0.125/≤0.06 | <0.25 | ≤0.5 | ≤0.06 |
| **D1_18 (V)** | 16 | 2 | 2 | ≤0.5 | ≤1/≤2 | ≤1 | 0.25 | ≤0.5 | ≤0.125 | 8 | ≤0.125/≤0.06 | >64 | ≤0.5 | ≤0.06 |
| **M1_15 (II)** | 4 | 2 | 1 | ≤0.5 | ≤1/≤2 | ≤1 | 0.5 | ≤0.5 | ≤0.125 | 8 | ≤0.125/≤0.06 | >64 | ≤0.5 | ≤0.06 |
| **D3_1 (V)** | 8 | 2 | 1 | ≤0.5 | ≤1/≤2 | ≤1 | 0.5 | ≤0.5 | ≤0.125 | 4 | ≤0.125/≤0.06 | >64 | ≤0.5 | ≤0.06 |
| **D3_24 (IV)** | 4 | 2 | 1 | ≤0.5 | ≤1/≤2 | ≤1 | 0.25 | ≤0.5 | ≤0.125 | 8 | ≤0.125/≤0.06 | >64 | ≤0.5 | ≤0.06 |
| **D3_8 (IV)** | 8 | 4 | 2 | ≤0.5 | ≤1/≤2 | ≤1 | 0.25 | ≤0.5 | ≤0.125 | 8 | ≤0.125/≤0.06 | >64 | ≤0.5 | ≤0.06 |
| **D1_20 (III)** | 16 | 2 | 1 | ≤0.5 | ≤1/≤2 | ≤1 | 0.25 | ≤0.5 | ≤0.125 | 4 | ≤0.125/≤0.06 | 4 | ≤0.5 | ≤0.06 |
| **M3_1 (I)** | >32 | 2 | >4 | ≤0.5 | 16/>4 | 8 | 0.5 | 4 | ≤0.125 | 16 | ≤0.125/≤0.06 | >64 | 8 | 0.5 |
| **M3_10 (II)** | >32 | 8 | >4 | >32 | >64/>4 | >64 | >8 | >8 | ≤0.125 | 4 | 8/4 | >64 | ≤0.5 | ≤0.06 |
| **M3_12 (II)** | >32 | >16 | >4 | >32 | ≤1/≤2 | 16 | >8 | >8 | >32 | >16 | >32/>16 | >64 | 32 | 4 |
| **D3_11 (III)** | >32 | >16 | >4 | >32 | >64/>4 | >64 | >8 | >8 | >32 | >16 | >32/>16 | >64 | >64 | >64 |
| **D3_13 (III)** | >32 | >16 | >4 | >32 | >64/>4 | >64 | >8 | >8 | >32 | >16 | >32/>16 | >64 | >64 | >64 |
| **D3_16 (III)** | >32 | >16 | >4 | >32 | >64/>4 | >64 | >8 | >8 | >32 | >16 | >32/>16 | >64 | >64 | >64 |
| **M2_34 (I)** | >32 | >16 | >4 | >32 | >64/>4 | >64 | >8 | >8 | >32 | >16 | >32/>16 | >64 | >64 | >64 |
| **M3_18 (I)** | >32 | >16 | >4 | >32 | >64/>4 | >64 | >8 | >8 | >32 | >16 | >32/>16 | >64 | >64 | >64 |

[a] isolates selected on the heatmap (Figure S1);

[b] MRD: Metronidazole, LZD: Linezolid, MXF: Moxifloxacin ,TGC: Tigecycline, P/T4: Piperacillin/ Tazobactam constant 4, PIP: Piperacillin, PE : penicillin, VAN: vancomycin, CHL: Chloramphenicol, AUG2: Amoxicillin / Clavulanic Acid, CLI: Clindamycin, RIF: Rifampicin, IMI: Imipenem.
